# Progressive Learning of a Multimodal Classifier Accounting for Different Modality Combinations

**DOI:** 10.3390/s23104666

**Published:** 2023-05-11

**Authors:** Vijay John, Yasutomo Kawanishi

**Affiliations:** Guardian Robot Project, RIKEN, Seika-cho, Kyoto 619-0288, Japan; yasutomo.kawanishi@riken.jp

**Keywords:** multimodal learning, person classification, emotion classification, missing modality, multimodal portability, sensor fusion

## Abstract

In classification tasks, such as face recognition and emotion recognition, multimodal information is used for accurate classification. Once a multimodal classification model is trained with a set of modalities, it estimates the class label by using the entire modality set. A trained classifier is typically not formulated to perform classification for various subsets of modalities. Thus, the model would be useful and portable if it could be used for any subset of modalities. We refer to this problem as the multimodal portability problem. Moreover, in the multimodal model, classification accuracy is reduced when one or more modalities are missing. We term this problem the missing modality problem. This article proposes a novel deep learning model, termed KModNet, and a novel learning strategy, termed progressive learning, to simultaneously address missing modality and multimodal portability problems. KModNet, formulated with the transformer, contains multiple branches corresponding to different *k*-combinations of the modality set *S*. KModNet is trained using a multi-step progressive learning framework, where the *k*-th step uses a *k-modal* model to train different branches up to the *k*-th combination branch. To address the missing modality problem, the training multimodal data is randomly ablated. The proposed learning framework is formulated and validated using two multimodal classification problems: audio-video-thermal person classification and audio-video emotion classification. The two classification problems are validated using the *Speaking Faces*, *RAVDESS*, and *SAVEE* datasets. The results demonstrate that the progressive learning framework enhances the robustness of multimodal classification, even under the conditions of missing modalities, while being portable to different modality subsets.

## 1. Introduction

**Figure 1:**
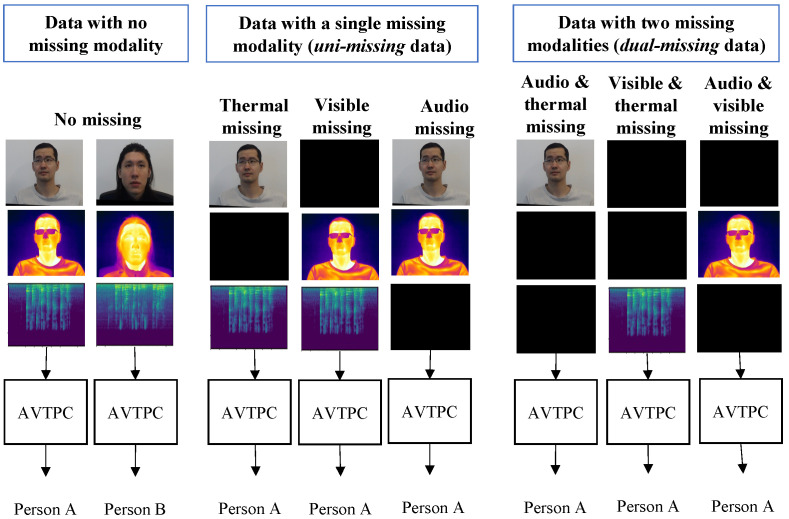
An overview of the missing modality problem in the audio-visible-thermal person classification (AVTPC) framework with *complete*, *uni-missing*, and *dual-missing* multimodal data.

**Figure 2:**
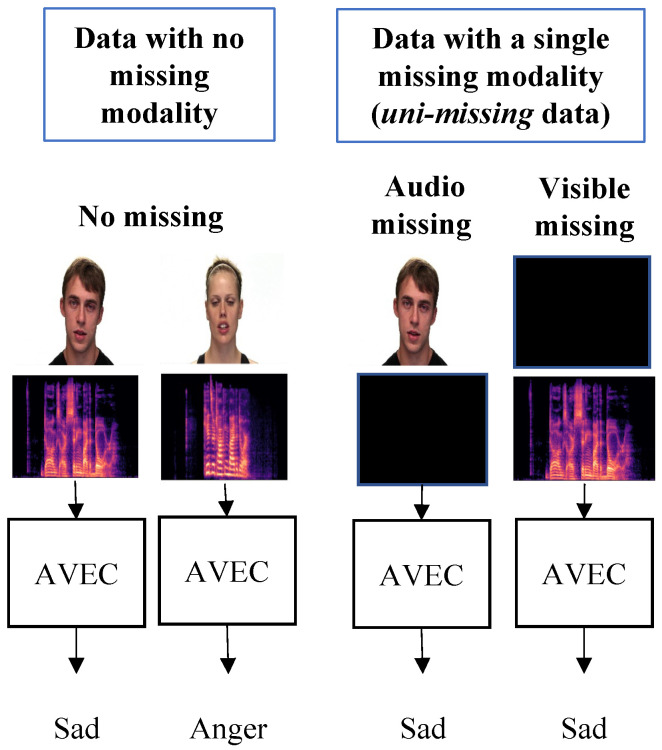
An illustration of the missing modality problem in the audio-visible emotion classification (AVEC) framework with *complete* and *uni-missing* multimodal data.

In classification tasks, such as face recognition and emotion recognition, multimodal information is often used to enhance classification accuracy and robustness. Multimodal classification addresses the limitations of state-of-the-art visible-camera-based classifications. These limitations include illumination and environmental variations, occlusions, background noise, and low light conditions. Multimodal classification addresses these limitations through the effective fusion of a visible camera with different sensors, such as a thermal camera [1,2,3,4,5]. However, given a set of *K* modalities, *S*, the multimodal classification framework typically relies on the availability of *complete* multimodal data for all modalities. Under the condition of *incomplete* multimodal data, where one or more modalities are missing, the performance of multimodal classification is affected [6,7,8]. This problem is referred to as the missing modality problem. Sensor failures, data corruption, and environmental noise are examples of scenarios resulting in *incomplete* multimodal data. For example, in the audio-visible emotion recognition problem, if the query person is outside the camera’s field of view, their visible camera appearance is not available and only audio data is available for classification. Similarly, in the case of loud background noise, audio data is not available, and only visible data is available. Illustrations of the missing modality problem are presented in Figure 1 and Figure 2.

For a given set *S* with *K* modalities, power set P(S) represents the set of all subsets of *S*. We define the set of the *k*-combination subsets, $S^{k}\in P\left(S\right)$, by selecting subsets of size *k* as

(1) $$\begin{array}{cc} S^{k} & =\left\{s\;|\;s\in \;P(S),|s|=k\right\}. \end{array}$$

**Figure 3:**
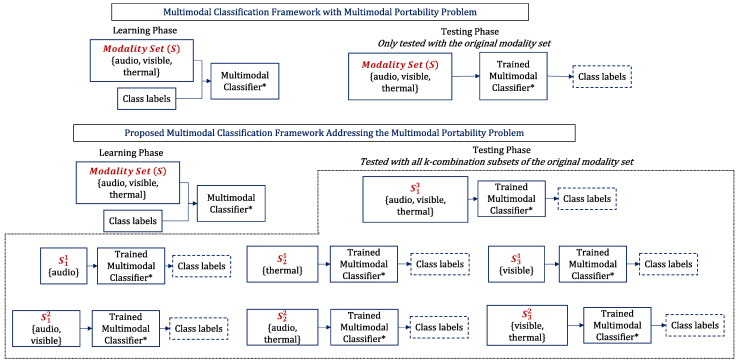
An overview of the multimodal portability problem. “Multimodal Classifier * represents the same classification model for the varying inputs”.

Typically, a multimodal classification framework is defined and trained using an original set of modalities. Subsequently, the trained framework is tested using the same modality set. This reduces the portability of the framework to different subsets, $S^{k}$. For example, given the set of {audio,visible,thermal} data, the 1-combination subsets $S^{1}={\left\{s_{i}^{1}\right\}}_{i=1}^{3}$ correspond to {audio}, {visible}, and {thermal}*unimodal* data. The 2-combination subsets $S^{2}={\left\{s_{i}^{2}\right\}}_{i=1}^{3}$ correspond to {audio,visible}, {visible,thermal}, and {audio,thermal}*bimodal* data. The 3-combination subset $S^{3}=\left\{s_{1}^{3}\right\}$ corresponds to {audio,visible,thermal} *trimodal* data. In this scenario, the standard *trimodal* classifier is often defined for *trimodal* data, and is not directly applied to *unimodal* or *bimodal* classification problems. We refer to this problem as the multimodal portability problem. An illustration of the problem is presented in Figure 3.

In this study, we propose a novel deep learning model, termed KModNet, which is trained with a novel progressive learning framework to simultaneously address missing modality and multimodal portability problems. KModNet is implemented with *K* blocks, with each *k*-th block containing different *k*-combination branches, where k≤K. KModNet is trained using a novel multi-step progressive learning framework, where each *k*-th step is used to train the different blocks in KModNet up to the *k*-th combination block. For example, the first step in the progressive learning framework is used to train the 1-combination block, and the second step is used to train the 1- and 2-combination blocks.

To enhance the robustness, in the *k*-th step, the 1 to (k−1) combination blocks trained in the previous steps are further fine-tuned. For example, the 1-combination block trained in the first step is further fine-tuned in the *k*-th step. To address the missing modality problem during training, multimodal data are randomly ablated to represent the missing modality data. Additionally, an “unknown” classification label is utilized to reduce the inefficient learning of certain models in the progressive learning framework. Finally, a multi-head attention transformer, which has been shown to be effective with missing modality data, is used [9].

The proposed learning framework is applied to two different classification tasks: audio-visible-thermal person classification (AVTPC) and audio-visible emotion classification (AVEC). The frameworks are validated using the *Speaking Faces* [10], *RAVDESS* [11], and *SAVEE* [12] datasets. The results demonstrate that the progressive learning framework enhances the robustness of multimodal classification, even under conditions of missing modalities, while being portable to different modality subsets. Owing to the formulations of KModNet with different *k*-combination blocks and the progressive learning strategy, the missing modality and multimodal portability problems are effectively addressed, as shown in the experimental section (Section 5).

The main contributions of this study to the literature are as follows:

- A novel multimodal classification framework termed the KModNet with *1* to *k*-combination blocks.
- A novel multimodal progressive learning framework to train the KModNet to address the missing modality and multimodal portability problems.

The remainder of this paper is organized as follows: The related literature is reviewed in Section 2. The proposed progressive learning framework is presented in Section 3, and its application for two classification tasks is presented in Section 4. The validation of the framework is performed in Section 5. Finally, we summarize and present our conclusions in Section 6.

## 2. Literature Review

Multimodal learning addresses the limitations of vision-based perception [4,5,13,14,15,16,17,18]. For example, an effective sensor fusion of the visible image with the audio and thermal image is shown to enhance the classification accuracy [2,3,4,5,13,19,20,21,22,23,24]. However, the aforementioned studies are limited by the missing modality and the multimodal portability problems.

In recent years, different approaches have been proposed to address the missing modality problem [7,8,25,26]. The different approaches can be categorized as generative, latent-space, data augmentation, and optimal fusion approaches. In the generative approach, the missing modality or supplementary data predicted from the available modality are used to enhance the classification accuracy. John et al. [27] proposed the audio-visible person classification framework, the CTNet, where the person label is estimated even when the visible image is missing. Here, person attributes, such as age, gender, and race, are predicted from the audio data. The person label is then estimated using the predicted attributes along with the audio data.

In the latent-space approach, the latent space is learned from multimodal data to address the missing modality problem [6,26,28,29]. Recently, John et al. [6] proposed a missing modality loss function to learn the latent space even under conditions of missing data. The latent space is used within the AVTNet to estimate the person class. Similarly, Zhao et al. [26] learned the latent representation from multimodal data using a residual autoencoder, which was subsequently used within the MMI network for emotion recognition.

In the data augmentation approach, researchers augment the training of multimodal data with ablated data, where the data corresponding to a missing modality are represented using pre-defined fixed data [6,30]. Finally, researchers have proposed optimal fusion frameworks using transformers to address the missing modality problem [9,31]. In the work by Ma et al. [9], a dataset-dependent fusion strategy was learned to enhance perception. Alternatively, Han et al. [31] adopted an implicit fusion strategy using multi-task learning, where the output layers were effectively shared by different modalities.

Although the aforementioned literature addresses the missing modality problem, it has not yet been solved. Moreover, the studies referred to also do not address the multimodal portability problem. In this article, compared to the literature, we propose a novel deep learning framework, the KModNet, and a novel progressive learning framework where we address the missing modality problem with multiple missing modalities in addition to the multimodal portability problem. In Table 1, we address the differences between the proposed algorithm and the related missing modality literature.

## 3. Proposed Classification Framework

### 3.1. Overview

For a set *S* with *K* modalities, powerset P(S) is the set of all subsets of *S*. The different *k*-combination subsets in P(S) are represented by the set $S^{k}\in P\left(S\right)$. Each $S^{k}$ contains $N_{k}$ elements that are given by $S^{k}$=${\left\{s_{i}^{k}\right\}}_{i=1}^{N_{k}}$.

The novel KModNet is formulated with multiple blocks corresponding to different $S^{k}\in P\left(S\right)$. Each *k*-th block $B^{k}$ contains $N_{k}$ branches corresponding to the different elements in $S^{k}$. Each *k*-combination branch $B_{i}^{k}$ accepts outputs only from the (k−1)-combination branches whose modalities are related to the modalities in $S_{i}^{k}$ as the input.

**Figure 4:**
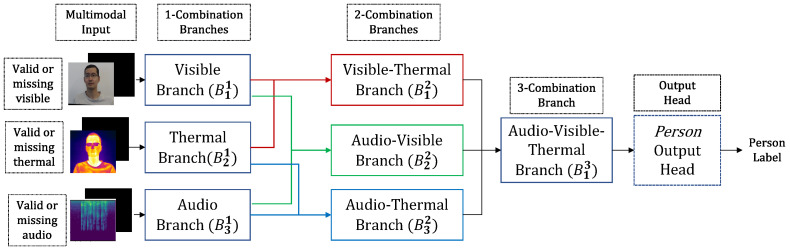
An example of the KModNet’s testing phase for the AVTPC problem.

The KModNet is trained using a multi-step progressive learning framework. Each *k*-th step in the progressive learning framework trains different blocks up to the *k*-th block in KModNet. Following the training, the different branches in KModNet can be used to estimate the class label for all *k*-combination subsets in P(S). An example of KModNet implemented for trimodal person classification is shown in Figure 4.

To handle the missing modality problem, first, the training multimodal data is randomly ablated using pre-defined fixed data representing the missing modality data. Next, to reduce inefficient learning owing to missing modality data, an “unknown” classification label is used along with the randomly ablated data for certain models in the progressive learning phase. These are explained in detail in subsequent sections. Finally, we utilize the multi-head attention-based transformer, which has been previously studied and shown to be effective for the missing modality problem [9].

### 3.2. Learning Phase

The progressive learning strategy is a multi-step learning framework where the different *k*-combination blocks in the KModNet are trained. Each *k*-th step in the learning strategy trains different blocks up to the *k*th-combination block in KModNet.

For each *k*-th step, 1−k combination blocks followed by a classification output head are used to train the network. In the first step, the 1-combination branches in the 1-combination blocks corresponding to $S^{1}$=${\left\{S_{i}^{1}\right\}}_{i=1}^{N_{1}}$ are trained independently. Here, the *i*-th deep learning model, followed by an individual classification head, is used to train the *i*-th 1-combination branch $B_{i}^{1}$ in block $B^{1}$. These models are termed *unimodal* models.

In the subsequent *k*-steps, a single deep learning model called the *k-modal* model is used to train the *k*-combination branches in the *k*-combination block, $B^{k}={B_{i}^{k}}_{i=1}^{N_{k}}$, corresponding to $S^{k}$ = ${\left\{S_{i}^{k}\right\}}_{i=1}^{N_{k}}$. In the *k-modal* model, $N_{k}$ classification heads are used to train the $N_{k}$ different *k*-combination branches.

In the *k-modal* model, first, to further enhance robustness, previously trained (k−1)−modal models without their classification heads are transferred. Next, the *i*-th *k*-combination branch $B_{i}^{k}$ in *k*-th combination block $B^{k}$ outputs a fused feature from the set of modalities in $S_{i}^{k}$. This branch selectively accepts the output of some of the branches in the previous block. The input of the *i*-th *k*-combination branch $B_{i}^{k}$ is represented by a set of outputs from the branches in the previous blocks, given as $\left\{B_{j}^{k-1}\;|\;S_{j}^{k-1}\cap S_{i}^{k}\neq Ø\right\}$. This is referred to as the *input-selection* mechanism.

Following the input selection, the transferred (*k*–1)−modal branches are also trained along with the *k*-combination branches. For example, in the case of bimodal KModNet, in the second step, the transferred *unimodal* models are also trained along with the 2-combination branches.

**Figure 5:**
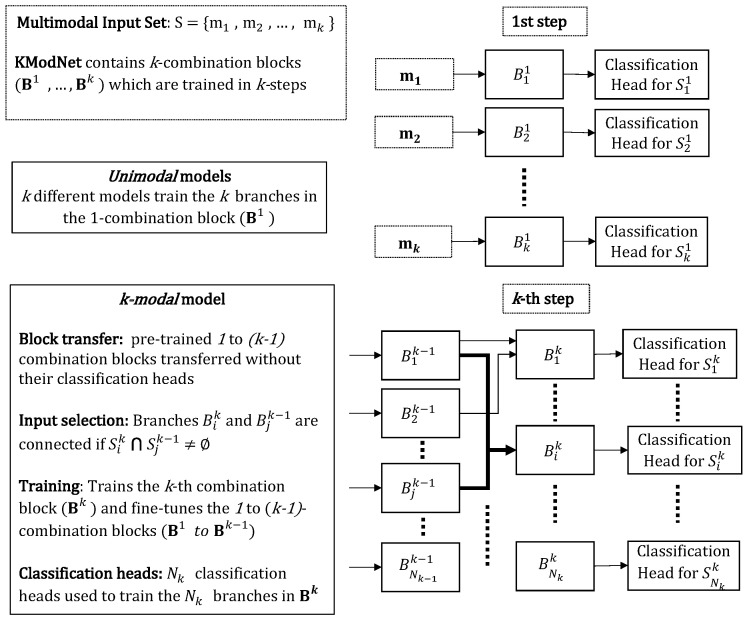
An overview of the progressive learning framework.

To address the missing modality data, in the learning phase, the multimodal data is randomly ablated with pre-defined fixed data. For multimodal data, if all modalities are present, the multimodal data are referred to as “complete” data. For trimodal data, if a single modality is missing, the data are referred to as “uni-missing” data. If two modalities are missing, the data are referred to as “dual-missing” data. The data corresponding to the missing modality is represented by pre-defined fixed data, the details of which are presented in Section 5. An overview of the KModNet learning phase is shown in Figure 5.

### 3.3. Testing Phase

Following progressive learning, the trained KModNet is used to estimate the classification label for the test multimodal data, even in the presence of missing modalities. Moreover, the trained *unimodal*, *bimodal*, and *k-modal* models in different learning steps can be used to estimate the class labels for different *k*-combinations of modality set *S*. Consequently, different branches in KModNet can be ported to any subset in P(S). This addresses the multimodal portability problem.

As shown in Section 5, the missing modality problem is effectively addressed because of the random ablation of the training data, use of the “unknown” classification labels for certain training steps, and integration of the transformers.

### 3.4. KModNet Implementation: Modal Specific

While the *k*-combination branches are generic, as their inputs are obtained from the preceding (k−1)-combination branch, the 1-combination branches should be designed for their corresponding modality because the multimodal data is given as input directly to KModNet’s 1-combination block, which represents the 1-combination subsets $S^{k}$. Here, we explain the modal-specific implementation of the 1-combination block.

In this article, KModNet is implemented for audio, visual, and thermal data, but not limited to them. The multimodal data for these tasks are represented by the audio spectrogram X, the video Y with *j* frames of size (64×64×3), and thermal video Z with *j* frames of size (64×64×1).

For visible and thermal videos, each image in the visible or thermal video sequence is split into 8×8 fixed-size patches and linearly embedded into a 128-dim projection space. Subsequently, learnable position embeddings are added to the linear embedding of each frame. The image embeddings are then concatenated to form a sequence vector. Frame embedding, which functions as a frame index, is added to the sequence vector. Frame embedding distinguishes embedded vectors among the three frames in the input video sequence. We refer to the aforementioned layers of patch extraction, position embedding, and frame embedding as *standard* embedding layers.

For the audio data, the audio spectrogram is given as input to three layers of Conv-1D with 32,64, and 128 filters of size 11,11, and 3, stride 1, and ReLU activation. We refer to this architecture as the *audio convolution* layers.

### 3.5. KModNet Implementation: Multi-Head Attention Transformer

Each branch in KModNet is implemented using a multi-head attention transformer. The layers in the transformer are termed *standard* transformer layers. Given the transformer input, the *standard* transformer layers have an initial layer normalization layer, followed by a multi-head attention layer with four heads and 0.1 drop out. The output of the attention layer is added to the input to obtain the attention output vector. This vector is given as an input to two multilayer perceptron (MLP) layers with 256 and 128 units with 0.1 dropout. The MLP output is then added to the attention output vector to obtain the output of the transformer branch.

For the 1-combination block, the inputs to the visible and thermal *standard* transformer layers in the visible and thermal-*unimodal* models correspond to the outputs of the *standard* embedding layers. For the audio *standard* transformer layers, the input to the audio-*unimodal* model is the output of the *audio* convolution layers.

For the *k*-combination blocks, the inputs to the *k-modal* model’s *standard* transformer layers are obtained by concatenating the outputs of certain branches in the (k−1)-combination block. The branches in the (k−1)-combination block are selected according to the previously described *input selection* mechanism.

## 4. Application of the Proposed Classification Framework

In this article, KModNet is formulated for two classification tasks: audio-visual-thermal person classification (AVTPC) and audio-visible emotion classification (AVEC).

### 4.1. AVTPC Problem

The KModNet for the AVTPC problem is trained to identify people using an audio-visible-thermal camera dataset $D_{t}={\left\{X_{i},Y_{i},Z_{i},l_{i}\right\}}_{i=1}^{U_{t}}$ with $U_{t}$ samples, where $X_{i}$ represents the audio, $Y_{i}$ represents the video with *j* frames, $Z_{i}$ represents the thermal video with *j* frames, and $l_{i}$ represents the person label. The audio input $X_{i}$ is represented by the Log-Mel-spectrogram of size 128×889 obtained from the audio using a sampling rate of 44,000 Hz. Video input $Y_{i}$ is represented by three uniformly sampled frames of size (64×64×3). Thermal input $Z_{i}$ is represented by three uniformly sampled frames of size (64×64×1). The video and thermal sequences are synchronized in the dataset.

#### 4.1.1. Classification Model

KModNet is formulated with three blocks with the corresponding *k*-combination branches. The first block contains three 1-combination branches, the second block contains three 2-combination branches, and the final block contains one 3-combination branch.

In the first block, in the visible ($B_{1}^{1}$) and thermal branches ($B_{2}^{1}$), visible and thermal sequences are provided as inputs to the *standard embedding* layers (Section 3.4). In the audio branch ($B_{3}^{1}$), the audio spectrogram is provided as an input to the *audio convolutional* layers. The output of these layers is then provided as an input to an individual *standard transformer* layer (Section 3.5).

In the second block, the outputs of the selective *standard transformer* layers are concatenated using the *input selection* mechanism and are provided as inputs to the specific *standard transformer* layers. For example, the outputs of the visible ($B_{1}^{1}$) and thermal ($B_{2}^{1}$) branches are concatenated and provided as inputs to the visible-thermal branch $\left(B_{1}^{2}\right)$. Similarly, the audio-visible $\left(B_{2}^{2}\right)$ and audio-thermal $\left(B_{3}^{2}\right)$ branches are implemented using individual *standard transformer* layers, the input of which is obtained by selective concatenation of the different branches in the first block.

Similar to the other blocks, the 3-combination branch ($B_{1}^{3}$) in the final block is also implemented using *standard transformer* layers. Here, the transformer inputs correspond to the concatenated outputs of the three 2-combination branches. The output of this block is given as input to the classification head, which contains a dense layer with 128 neurons, batch normalization, and a ReLU activation unit, followed by a dense layer with 143 neurons with a softmax activation function. Hereafter, we refer to the output head as the *person* classification head.

The three blocks are progressively trained in three steps using the *unimodal*, *bimodal*, and *trimodal* models. Following training, KModNet estimates the person label for the *complete* data, “uni-missing”, and “dual-missing” data. Trained *unimodal* and *bimodal* models can be used for different *k*-combination subsets to address the multimodal portability problem. An overview of the testing phase of the KModNet is shown in Figure 4.

#### 4.1.2. Progressive Learning

In the first step, the 1-combination branches in the 1-combination block representing the audio, visible camera, and thermal camera data are trained using three *unimodal* models. The visible and thermal *unimodal* models contain *standard embedding* layers (Section 3.4), *standard transformer* layers (Section 3.5), and a *person* classification head. The audio unimodal model contains *audio convolution* layers, *standard transformer* layers (Section 3.5), and a *person* classification head.

In the second step, a deep learning model termed the *bimodal* model, is used to train the three 2-combination branches in the 2-combination block using three *person* classification heads in a multi-task formulation. The 2-combination branches represent the audio-visible, audio-thermal, and visible-thermal camera data. In the *bimodal* model, the three trained *unimodal* models without their classification heads are first transferred to the bimodal model. Subsequently, each 2-combination branch implemented by the *standard transformer* layers accepts the outputs of specific *unimodal* branches. The audio-visible branch accepts the outputs of the audio and visible *unimodal* branches. The audio-thermal branch accepts the outputs of the audio and thermal *unimodal* branches. The visible-thermal branch accepts the outputs of the visible and thermal *unimodal* branches.

Each 2-combination branch has an individual *person* classification output head resulting in a multi-task formulation. During the learning phase, the *bimodal* model trains the pre-trained *unimodal* models along with the 2-combination branches.

**Figure 6:**
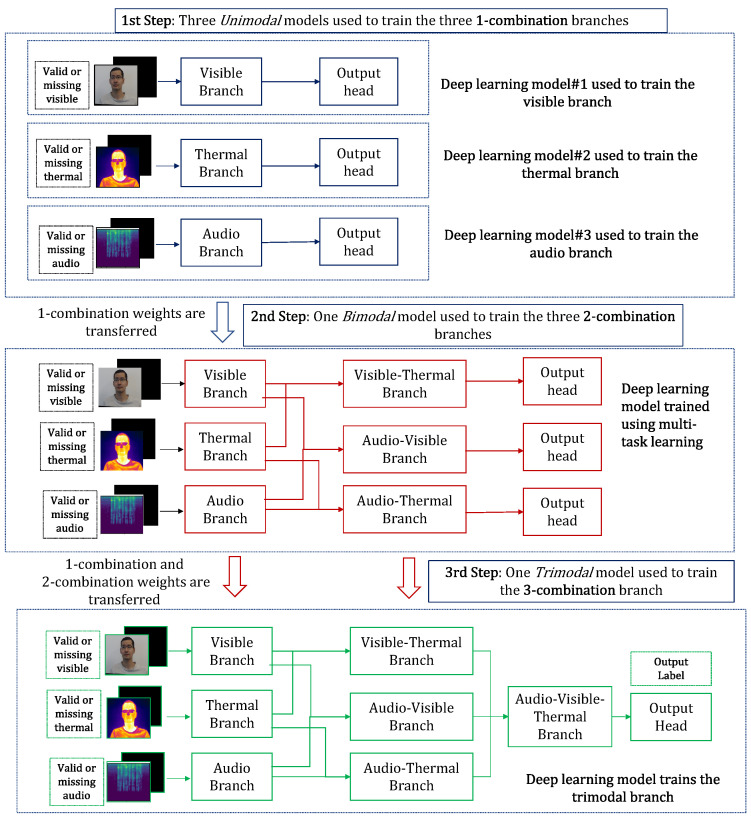
Progressive learning for the AVTPC problem.

In the third step, a deep learning model, termed the *trimodal* model, is used to train a single 3-combination branch in the 3-combination block representing the audio-visible-thermal camera data. Similar to the previous step, the pre-trained *bimodal* model is first transferred to the *trimodal* model without the three classification heads. Next, the 3-combination branch implemented by the *standard transformer* layers accepts the outputs of all the *bimodal* branches. Finally, a single *person* classification head is used to train the 3-combination branch along with the transferred 1- and 2-combination branches. An overview of the learning phase is shown in Figure 6.

To eliminate inefficient learning owing to missing training data at the output head, the “unknown” person label is included in addition to the person labels for training the *unimodal* and *bimodal* models. In the case of the *trimodal* model, the output head does not receive training data with missing modalities. Consequently, the “unknown” person label is not added to the person labels.

### 4.2. AVEC Problem

The KModNet used for the AVEC problem is trained to identify emotions using an audio-visible camera dataset $D_{e}={\left\{X_{i},Y_{i},l_{i}\right\}}_{i=1}^{S}$ with $U_{e}$ samples, where $X_{i}$ represents the audio, $Y_{i}$ represents the video with *j* frames, and $l_{i}$ represents the emotion label. The audio input $X_{i}$ is represented by the Log-Mel-spectrogram image of size 128×889. Video input $Y_{i}$ is represented by three frames of size (64×64×3).

#### 4.2.1. Classification Model

**Figure 7:**
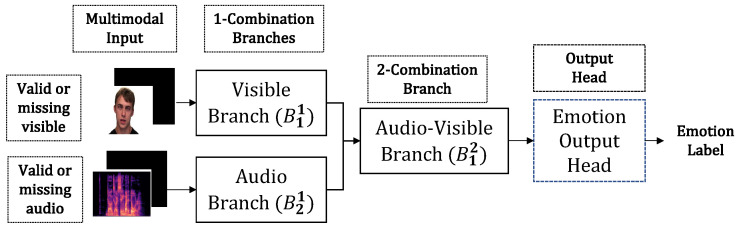
An overview of the KModNet’s testing phase for the AVEC problem.

For the bimodal classification problem, KModNet contains two blocks with two 1-combination branches and one 2-combination branch (Figure 7). In the visible branch of the first block ($B_{1}^{1}$), visible sequences are provided as inputs to the *standard embedding* layers (Section 3.4). In the audio branch ($B_{2}^{1}$), the audio spectrogram is provided as an input to the *audio convolutional* layers (Section 3.4). The output of these layers is then provided as an input to an individual *standard transformer* layer (Section 3.5).

The second block is implemented using *standard transformer* layers. Here, the outputs from the two 1-combination branches are concatenated and provided as inputs to the transformer. The output of the second block is given as input to the classification head, which contains a dense layer with 128 neurons, batch normalization, and a ReLU activation unit, followed by a dense layer with *O* neurons with a softmax activation function. The number of output neurons *O* varies with the dataset. Hereafter, we refer to the output head as the *emotion* classification head.

The different *k*-combination branches are trained progressively in two steps using the *unimodal* and *bimodal* models. Following training, KModNet estimates the emotion label for *complete* data and “uni-missing” data. Moreover, trained *unimodal* models can be used for 1-combination modalities. An overview of the testing phase of the KModNet is shown in Figure 7.

#### 4.2.2. Progressive Learning

In the first step, the 1-combination branches representing the audio and visible camera data are individually obtained using a *unimodal* model. The visible *unimodal* model contains *standard embedding* layers (Section 3.4), *standard transformer* layers (Section 3.5), and an *emotion* classification head. The audio *unimodal* model contains an *audio convolution* layer, *standard transformer* layers (Section 3.5), and an *emotion* classification head.

**Figure 8:**
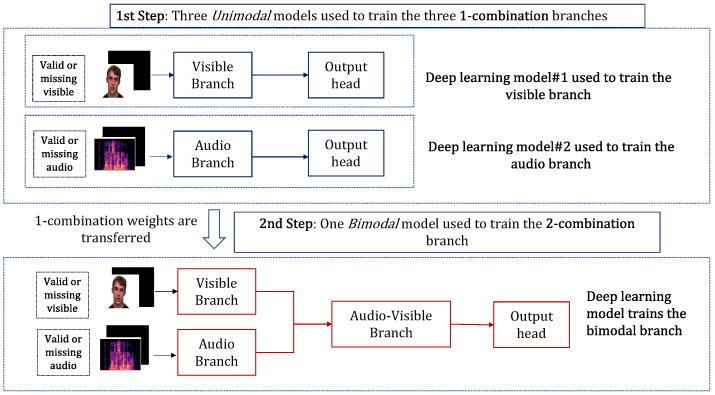
An overview of the progressive learning for the AVEC problem.

In the second step, the *bimodal* model is used to train the 2-combination branch. The 2-combination branch represents audio–visible camera data. Similar to the AVTPC problem, in the *bimodal* model, the two trained *unimodal* models, without their classification heads, are transferred to the bimodal model. Subsequently, the audio-visible branch, implemented with *standard* transformer layers, accepts the audio and visible *unimodal* branch outputs. The output of the transformer layers is provided as input to the *emotion* classification head. An overview of the progressive learning is shown in Figure 8.

## 5. Experiments

The proposed framework for the AVTPC problem is validated using the audio-visible-thermal Speaking Faces dataset [10]. Similarly, the proposed framework for the AVEC problem is validated using the audio-visible RAVDESS dataset [11] and the SAVEE dataset [12].

### 5.1. AVTPC Problem

#### 5.1.1. Dataset

In the Speaking Faces dataset, 3310 audio-visible-thermal sequences corresponding to 142 people are selected and randomly partitioned into the training and testing sequences. For the learning phase, we generate a missing dataset from the original dataset by randomly ablating 20% of the training data. For the 20% missing dataset, half of the sequences have ablated data corresponding to a single missing modality representing the *uni-missing* data, whereas the remaining half have ablated data corresponding to two missing modalities representing the *dual-missing* data.

The proposed framework is validated using multiple baseline algorithms. In addition, we perform a detailed validation of the progressive learning framework.

#### 5.1.2. Baseline Algorithms

The first baseline algorithm is formulated using the convolution neural network (CNN). The audio data are provided as input to the *audio convolution* branch, and audio feature maps are extracted. Next, each frame in the visible and thermal videos is given as input to three layers of Conv-2D with 32,64, and 32 filters of size 2, stride 2, and ReLU activation to extract the visible and thermal feature maps. The audio, visible, and thermal feature maps are then concatenated according to the classification problem and provided as input to the *person* output head.

The second baseline algorithm is formulated using the multi-head attention transformer [32]. The audio features are extracted using the *audio convolution* branch. Visible and thermal features are obtained using *standard* embedding layers. The audio, visible, and thermal features are then concatenated according to the classification problem and given as inputs to the *standard* transformer branch and the *person* output head. Hereafter, this baseline will be referred to as the transformer model.

The third baseline algorithm is formulated using the *standard* transformer branch and the missing modality loss proposed by John et al. [6]. Similar to the second baseline algorithm, audio, visible, and thermal features are extracted using the *audio convolution* branch and *standard* embedding layers. The individual latent spaces are first learned using a metric-learning-based missing modality loss with the extracted features. Next, the extracted features are concatenated, and the joint latent space is learned using the same loss function. Finally, the individual and joint latent spaces are used within a *k*-NN classifier to estimate the person label. Hereafter, we refer to this baseline as the latent model.

#### 5.1.3. Progressive Learning Validation

In this study, we validate the progressive learning framework by comparing the accuracies of the trained *unimodal*, *bimodal*, and *trimodal* models trained at different steps of the framework. Here, we utilize the trained models and their *person* classification heads (Section 4.1.2).

Additionally, we perform a comparative analysis of the two variants of the proposed KModNet. The first variant is an end-to-end framework (E2E-KModNet), where KModNet is trained directly in a single step without any multi-step progressive learning.

In the second variant, KModNet is trained using a multi-step learning strategy, but the *unimodal*, *bimodal*, and *trimodal* branches are trained only once. More specifically, following the training of the 1-combination branches in the first step, their weights are frozen and transferred to the *bimodal* model. In the second step, only the 2-combination branches are trained, and the 1-combination branches are not fine-tuned. In the case of the AVTPC problem, in the third step, only the 3-combination branches are trained without any fine-tuning of the other branches. Hereafter, we refer to the second variant as the DT-KModNet.

#### 5.1.4. Training Parameters

The proposed frameworks are implemented with TensorFlow 2 using NVIDIA 3090 GPUs on an Ubuntu 20.04 desktop. The different deep learning models and baseline algorithms are trained for 50 epochs, except for the multi-tasking *bimodal* model and third baseline models (latent space algorithm), which are trained for 100 epochs. The deep learning models are trained at a learning rate of 0.001, $\beta_{1}$ = 0.5, and $\beta_{2}$ = 0.99. The training parameters are empirically selected and represent the best performances of the different algorithms.

#### 5.1.5. Experimental Results

The performance of the proposed, the baseline, and the ablation study variants are reported in Table 2 and Table 3. In the testing phase, we report the classification accuracy for different ablations of the testing sequences. The ablations are represented by replacing the modality data with pre-defined fixed data. Apart from the original data, the test ablations include the *uni-missing* and *dual-missing* data.

The comparative results of classification accuracies are listed in Table 2. The results show that both the proposed frameworks have better accuracy than the baseline algorithms.

The results of the progressive learning validation are shown in Table 3. The results clearly demonstrate the advantage of the multi-step learning framework used within the proposed frameworks.

### 5.2. AVEC Problem

#### 5.2.1. Dataset

In the SAVEE dataset, 480 audio-visible sequences from four people with seven different emotions are selected. In the RAVDESS dataset, 1440 sequences from 24 actors with eight emotions and two trials are selected. The SAVEE dataset is randomly partitioned into training and testing sequences, whereas in the RAVDESS dataset, the first trial sequences are used for training, and the second trial sequences are used for testing. In the learning phase, the missing dataset is generated from the original data by randomly ablating 20% of the training sequences to obtain *uni-missing* data. The ablated visible and thermal images are represented by zero images of size 64×64×3 and 64×64×1. In contrast, ablated audio is represented by a zero image of size 128×889.

#### 5.2.2. Baseline Algorithms

For the AVEC validation, in addition to the baseline algorithms used for the AVTPC problem, the following AVEC algorithms by John et al. [33], Ristea et al. [34], and Mandeep et al. [35] are also used for the comparative analysis.

For progressive learning validation, similar to the AVTPC problem, we validate the progressive learning framework by comparing the accuracy of the trained *unimodal* and *bimodal* models with their *emotion* classification heads (Section 4.2.2).

In addition, a comparative analysis is performed using the two variants of the proposed framework. The first variant is an end-to-end framework (E2E-KModNet), in which the *bimodal* model is directly trained in a single step without any pre-training. In the second variant, multi-step learning is utilized, but the *unimodal* branches are trained only once. More specifically, following the training of the 1-combination branches in the *unimodal* model, their weights are frozen and transferred to the *bimodal* model. Henceforth, we refer to the second variant as the DT-KModNet framework.

#### 5.2.3. Training Parameters

The different deep learning models and baseline algorithms are trained for 50 epochs, except for the third baseline model (latent-space algorithm), which is trained for 100 epochs. The deep learning models are trained at learning rates of 0.001, $\beta_{1}$ = 0.5, and $\beta_{2}$ = 0.99. The training parameters are empirically selected and represent the best performances of the different algorithms.

#### 5.2.4. Experimental Result

The performances of the proposed, baseline, and variants are reported in Table 4, Table 5, Table 6 and Table 7. In the testing phase, we report the classification accuracy for different ablations of the testing sequences. The ablations are represented by replacing the modality’s data with pre-defined fixed data. In addition to the original data, the test ablations include *uni-missing* data.

The comparative results for emotion classification accuracy are listed in Table 4 and Table 5. The results show that both the proposed frameworks have better accuracy than the baseline algorithms.

The results of the progressive learning validation are shown in Table 6 and Table 7. The results clearly demonstrate the advantage of the multi-step learning framework used within the proposed frameworks.

### 5.3. Discussion

**Missing Modality:** The missing modality problem is observed in the results, where the performance drops with the single missing modality. The performance of the original test dataset, without any missing modality, was the best. Moreover, in Table 3, we observe further degradation of accuracy when the two modalities are missing.
**Comparison with Baseline Models:** The advantages of the proposed framework are observed in Table 2, Table 4 and Table 5 where the progressive framework reports a better classification accuracy than baseline algorithms while addressing the missing modality problem. In addition, unlike the different baseline algorithms, the proposed framework addresses the multimodal portability problem.

Among the different baseline methods, for the AVTPC problem (Table 2), the transformer-based baseline models, including the transformer model, E2E, and DT, report better results than the CNN-based baseline models. In the case of the SAVEE dataset results of the AVEC problem (Table 5), the CNN and transformer-based baseline models, on average, report similar accuracies. On the other hand, in the case of the RAVDESS dataset (Table 4), the performance of certain CNN and transformer-based baseline models, such as the CNN, E2E, DT, and Ristea et al. [34] are similar and higher than those of the remaining CNN and transformer-based baseline models. On average, the transformer-based models report better accuracy than the CNN-based models, which is similar to observations in the literature [9,32].

The latent-space-based baseline model by John et al. [6] reports a good accuracy for the AVTPC problem and SAVEE of the AVEC problem’s SAVEE; however, in the case of the RAVDESS dataset, the latent model does not perform well. The latent-space model is only formulated for occasionally missing data in an individual modality, and not for a missing modality in multimodal data [6]. Based on these results, we can observe that the performance of the different baseline algorithms is dataset-dependent.

**Validation of the Progressive Learning Framework:** In this paper, in the novel progressive learning framework, the multi-step learning and the fine-tuning of the previously trained branches are important contributions to the literature. We validate these contributions using the DT and E2E variants of the algorithms. The results in Table 2, Table 4 and Table 5 show the advantages of multi-step learning, as the proposed framework yields better results than E2E-KModNet, which corresponds to the single-step trained KModNet.

The results also demonstrate the advantages of fine-tuning the previously trained branches within the progressive learning framework because the proposed framework has better accuracy than the DT-KModNet model. Unlike the proposed learning framework, in DT-ModNet, the pre-trained branches transferred from the preceding block are not fine-tuned.

The advantages of the progressive learning framework are listed in Table 3, Table 6 and Table 7. Here, a layer-wise increase in classification accuracy across different models can be clearly observed. This can be attributed to the sensor fusion of the different *k*-combinations and the progressive learning framework, where the previously trained branches are fine-tuned.

The results of the 1-combination or *unimodal* models are obtained as averages of the different *unimodal* models. In the case of AVEC, the *bimodal* model yields better results than the *unimodal* models (Table 6 and Table 7).

In the case of AVTPC, the results of the *bimodal* and *unimodal* models are obtained by averaging the accuracies of the different output heads. It can be observed that the *trimodal* models report better accuracy than the *bimodal* models, which, in turn, report better accuracy than the *unimodal* models. The progressive improvement in accuracy can be attributed to sensor fusion and fine-tuning of the previously trained branches.

**Generalization across Varying Inputs:** Compared to the baseline algorithms, the proposed algorithm reports the best results across the different types of input data, the *complete*, the *uni-missing*, and the *dual-missing* data, as demonstrated by the average classification accuracy. For example, in Table 5, the third baseline algorithm (Latent) [6] provides better accuracy for *complete* and *audio-missing* data but does not classify *visible-missing* data accurately. This result can be attributed to the latent baseline algorithm overfitting *complete* and *audio-missing* data during the learning phase. However, the proposed algorithm can learn across different types of missing and complete data without overfitting any missing data.
**Multimodal Portability:** The multimodal portability of the proposed framework is observed in the ablation study, where the *unimodal*, *bimodal*, and *trimodal* with their *person* and *emotion* classification heads can be easily ported to a different *k*-combination of modalities (Section 4.1.2 and Section 4.2.2).
**Varying Modalities:** In multimodal learning, a given modality can be a dominant or a weaker modality. The experimental results for the AVTPC problems show that the audio modality is weaker than the visible and thermal modalities (Table 2). This is observed in the results of the *visible-thermal* missing data, where only audio is present, indicating a low classification accuracy. However, the visible camera is shown to be the dominant modality, as observed in the *unimodal* missing data, where the *visible missing* data report inferior results compared to the *thermal missing* and *audio missing* data.

In the case of the AVEC problem, modality characteristics are dataset-dependent. In the RAVDESS dataset (Table 4), the audio and visible modalities have similar strengths, with the audio being marginally dominant, as the *visible missing* data report better accuracy than the *audio missing* data. However, in the case of the SAVEE dataset (Table 5), audio is the weaker modality because the *visible missing* data are inferior to the *audio missing* data.

Based on the results, we can conclude that certain modalities are either dominant or weaker depending on the situation; thus, their effective sensor fusion in progressive learning enhances the robustness of classification tasks.

**Future work:** In the results, we can observe that the absence of the dominant modality, the visible camera, reduces the classification accuracy (Table 4 and Table 5). As part of our future work, we will investigate and consider multimodal co-learning techniques [36] to ensure that all modalities contribute equally to learning. Specifically, we will focus on the conditions under which the dominant modality is missing.

In the progressive learning framework, different *k*-combinations can be trained using an additional unimodal or bimodal dataset. For example, in the AVEC framework, in addition to training with the bimodal dataset, *unimodal* models can also be trained with an audio-only or visible camera-only emotion classification dataset. The advantages of additionally training a unimodal or bimodal model with a modality-specific dataset will be investigated in future work.

## 6. Conclusions

In this article, a novel progressive learning framework is proposed to train a deep learning framework with multiple *k*-combination blocks, termed KModNet, to address the missing modality and multimodal portability problems. Progressive learning is a multi-step learning strategy formulated to train different *k*-combination blocks in KModNet. Each *k*-th step in the learning strategy is formulated to train all *k*-combination blocks up to the *k*-th combination block. Multiple deep learning models were used in the learning strategy. By accounting for the different *k*-combinations in KModNet and utilizing the progressive learning strategy, we simultaneously address the missing modality and multimodal portability problems. We validate the proposed learning strategy using two multimodal classification tasks: person classification and emotion classification. The frameworks are validated using the *Speaking Faces*, *RAVDESS*, and *CREMA* datasets. The results and ablation study demonstrate that the progressive learning framework enhances the robustness of multimodal classification, even under the conditions of missing modalities, while being portable to different modality subsets.

## Figures and Tables

**Table 1:** Comparison of the related literature addressing the missing modality problem (MMP). MPP represents the multimodal portability problem.

Algo	Research	Modalities	No. of Missing	MMP Approach	Address
	Problem		Modalities in		MPP
			Data		
Prop.	Person &	Aud.-Vis.-Th.	Single	Data Augment. &	Yes
	Emotion Class.	Aud.-Vis.	& Double	Prog. Learning	
Ma et al. [25]	Multilabel,	Aud.-Vis.	Single	Generative	No
	Binary, &	Aud.-Vis.-Txt.			
	Multiclass Class.	Vis.-Txt.			
Zhao et al. [26]	Emotion Class.	Aud.-Vis.-Txt.	Single &	Latent Space &	No
			Double	Generative	
Yang et al. [7]	FAU Class.	Vis.-Th.-Depth.	Missing	Opt. Fusion	No
			Patches		
Cai et al. [8]	Disease Det.	PET-MRI	Single	Generative	No
John et al. [27]	Person Class.	Aud.-Vis.	Single	Generative	No
Pham et al. [28]	Sentiment Pred.	Aud.-Vis.-Txt.	Single &	Latent Space	No
			Double		
John et al. [6]	Person Class.	Aud.-Vis.-Th.	Single	Latent Space &	No
				Data Augment.	
Parthasarathy et al. [30]	Express Class.	Aud.-Vis.	Single	Data Augment.	No
Han et al. [31]	Emotion Class.	Aud.-Vis.	Single	Opt. Fusion	No
Ma et al. [9]	Multiclass Class.	Vis.-Txt.	Single	Opt. Fusion	No

**Table 2:** Comparative analysis of the average person classification accuracy for the Speaking Face dataset. Bold accuracy represents the best classification accuracy.

Algo	No Miss.	Aud. Miss.	Vis. Miss.	Th. Miss.	Vis-Th.	Aud-Th.	Aud-Vis.	Avg
					Miss.	Miss.	Miss.	
Prop	**98.64**	**95.77**	87.46	**97.20**	35.20	**88.51**	68.70	**81.64**
CNN	87.91	87.61	39.27	46.1	2	54.5	52.7	52.87
Trans. [32]	90.1	90.1	56.49	70.99	2	70.99	60.1	62.96
Latent [6]	95.1	91.1	85.2	86.5	18	77.1	**77.5**	75.7
E2E-KModNet	98.79	86.7	**93.3**	79.15	25.5	42.74	63.89	70
DT-KModNet	97.3	87.6	86.7	92.74	**40.4**	70.84	54.53	75.7

**Table 3:** Validation of the progressive framework for the Speaking Face dataset. Bold accuracy represents the best classification accuracy.

Algo	No Miss.	Aud. Miss.	Vis. Miss.	Th. Miss.	Vis-Th.	Aud-Th.	Aud-Vis.	Avg
					Miss.	Miss.	Miss.	
Unimodal	87.83	59.5	56.69	59.86	28.49	31.36	28.19	50.27
Bimodal	97.33	89.1	79.4	82.83	**42.46**	59.13	52.41	71.80
Trimodal	**98.64**	**95.92**	**87.91**	**97.58**	36.55	**88.67**	**68.58**	**81.97**

**Table 4:** Comparative analysis of the average emotion classification accuracy for the RAVDESS dataset. Bold accuracy represents the best classification accuracy.

Algo	No Miss.	Aud. Miss.	Vis. Miss.	Avg
Prop	**82.36**	42.77	59.02	**61.38**
CNN	73.75	48.61	40.55	54.30
Transformer. [32]	51.3	40.83	23.8	38.64
Latent [6]	54.5	30.13	15.4	33.34
E2E-KModNet	73.3	47.91	44.30	55.17
DT-KModNet	80.83	38.05	**62.08**	60.32
John et al. [33]	66.9	29.0	41.6	45.83
Ristea et al. [34]	69.7	37.36	47.22	51.42
Mandeep et al. [35]	52.7	**52.7**	13.3	39.56

**Table 5:** Comparative analysis of the average emotion classification accuracy for the SAVEE dataset. Bold accuracy represents the best classification accuracy.

Algo	No Miss.	Aud. Miss.	Vis. Miss.	Avg
Prop	81.66	78.33	44.16	**68.05**
CNN	88.33	79.16	25.83	64.44
Transformer. [32]	85.83	87.5	29.16	67.49
Latent [6]	**89.16**	**90**	19.16	66.10
E2E-KModNet	89.1	82.5	30.8	67.46
DT-KModNet	80.83	68.3	**49.16**	66.09
John et al. [33]	65	42.5	21.6	43.03
Ristea et al. [34]	86.6	76.6	29.1	64.1
Mandeep et al. [35]	85.8	85.83	14.16	61.93

**Table 6:** Validation of the progressive framework on the RAVDESS dataset. Bold accuracy represents the best classification accuracy.

Algo	No Miss.	Aud. Miss.	Vis. Miss.	Avg
Unimodal	64.55	**43.7**	20.8	43.01
Bimodal	**81.2**	40.7	**57.9**	**59.93**

**Table 7:** Validation of the progressive framework on the SAVEE dataset. Bold accuracy represents the best classification accuracy.

Algo	No Miss.	Aud. Miss.	Vis. Miss.	Avg
Unimodal	65.4	43.75	21.65	43.01
Bimodal	**85**	**79.1**	**43.3**	**59.93**

## Data Availability

Not applicable.
